# Experience in Managing Severe Malnutrition in a Government Tertiary Treatment Facility in Bangladesh

**DOI:** 10.3329/jhpn.v27i1.3319

**Published:** 2009-02

**Authors:** Md. Iqbal Hossain, Nina S. Dodd, Tahmeed Ahmed, Golam Mothabbir Miah, Kazi M. Jamil, Baitun Nahar, Badrul Alam, C.B. Mahmood

**Affiliations:** ^1^ ICDDR,B, Mohakhali, Dhaka 1212, Bangladesh; ^2^ Concern Worldwide Bangladesh, House 58, 1^st^ Lane, Kalabagan, Dhaka 1205, Bangladesh; ^3^ Chittagong Medical College Hospital, Chittagong, Bangladesh

**Keywords:** Child nutrition, Child nutrition disorders, Healthcare cost, Infant nutrition, Infant nutrition disorders, Oral rehydration therapy, Severe acute malnutrition, Standardized protocol, Bangladesh

## Abstract

Children with severe acute malnutrition, defined as weight-for-height <70% of the reference median or bilateral pedal oedema or mid-arm circumference <110 mm having complications, were managed in the Nutrition Unit of the Chittagong Medical College Hospital (CMCH) following the guidelines of the World Health Organization, with support from Concern Worldwide Bangladesh and ICDDR,B. In total, 171 children aged less than five years (mean±SD age 23.5±15.3 months) were admitted during June 2005–May 2006. Of them, 66% were aged less than two years, and 84.2% belonged to households with a monthly income of less than US$ 40. The main reason for bringing children by their families to the hospital was associated major illnesses: bronchopneumonia (33%), oedema (24%), diarrhoea (11%), pulmonary tuberculosis (9%), or other conditions, such as meningitis, septicaemia, and infections of the skin, eye, or ear. The exit criteria from the Nutrition Unit were: (a) for children admitted without oedema, an absolute weight gain of ≥500 and ≥700 g for children aged less than two years and 2-5 years respectively; and for children admitted with oedema, complete loss of oedema and weight-for-height >70% of the reference median, and (b) the mother or caretaker has received specific training on appropriate feeding and was motivated to follow the advice given. Of all the admitted children, 7.6% of parents insisted for discharging their children early due to other urgent commitments while 11.7% simply left with their children against medical advice. Of the 138 remaining children, 88% successfully graduated from the Nutrition Unit with a mean weight gain of 10.6 g/kg per day (non-oedematous children) and loss of −1.9 g/kg per day (oedematous children), 86% graduated in less than three weeks, and the case-fatality rate was 10.8%. The Nutrition Unit of CMCH also functions as a training centre, and 197 health functionaries (82 medical students, 103 medical interns, and 12 nurses) received hands-on training on management of severe malnutrition. The average cost of overall treatment was US$ 14.6 per child or approximately US$ 1 per child-day (excluding staff-cost). Food and medicines accounted for 42% and 58% of the total cost respectively. This study demonstrated the potential of addressing severe acute malnutrition (with complications) effectively with minimum incremental expenditure in Bangladesh. This public-private approach should be used for treating severe acute malnutrition in all healthcare facilities and the treatment protocol included in the medical and nursing curricula.

## INTRODUCTION

More than 90% of the global burden of malnutrition is attributable to 36 countries of the world, and Bangladesh is one of them (1). Although Bangladesh is one of a few countries poised to achieve the United Nations' Millennium Development Goal (MDG) 1 (malnutrition component), the rates and absolute numbers of malnourished children are much higher than in sub-Saharan Africa (2). The target of MDG 4 for Bangladesh is to reduce mortality of children aged less than five years (under-five mortality) from 151/1,000 livebirths in 1990 to 50/1,000 livebirths in 2015. The rates of under-five and infant mortality for the 1999-2003 period were 88 and 65 per 1,000 livebirths respectively (3). This means that one in 11 children born in Bangladesh dies before reaching the fifth birthday while one in 15 dies before the first birthday. Children with severe acute malnutrition are more likely to fall sick and contribute to high mortality and morbidity. Although children are usually taken to a healthcare facility for acute illnesses, including pneumonia or tuberculosis, severe malnutrition is often an underlying causal factor, the importance of which is not well-recognized to warrant specific treatment (4). It is well-accepted that existing facilities in medical college hospitals are limited only to handle complicated diseases, e.g. pneumonia, and the result of this persistent oversight is clear: the treatment of severely-malnourished children with dehydration, pneumonia, and other infections is just similar to that of well-nourished children with these conditions resulting in disastrous consequences (5). Implementation of a standard hospital treatment protocol based on the guidelines of the World Health Organization (WHO) (6) is an essential first step for the facility-based management of severe acute malnutrition. It has been suggested that the mere presence of a standardized protocol increases discipline and attention to detail and results in fewer errors by health workers (7,8). As long as severe malnutrition is prevalent, efforts to improve its treatment and outcome remain a priority. With support from Concern Worldwide Bangladesh and ICDDR,B, the Chittagong Medical College Hospital (CMCH) established a Nutrition Unit for the management of children with severe acute malnutrition and for hands-on training of medical students, nurses, and doctors. The objective of this collaboration was to provide evidence-based results for policy-makers that management of children with severe acute malnutrition and complications in Bangladesh, using a protocolized treatment based on the guidelines of WHO, is feasible and effective.

## MATERIALS AND METHODS

Through a collaborative approach, the CMCH, Concern Worldwide Bangladesh, and ICDDR,B pooled resources to manage under-five children with severe acute malnutrition and associated complications at the Nutrition Unit of CMCH. The CMCH was responsible for the implementation of this initiative by arranging the basic infrastructure (space for the Nutrition Unit and beds), medicines, food, logistics, necessary financial allocations, ongoing operational costs as per the government rules, and staff to run the unit and for supervision and monitoring the activities. Concern Worldwide Bangladesh recruited one Nutrition Counsellor and one Medical Officer for daily monitoring of the initiative and provided weighing scales and additional cost of medicines, special foods, and stationeries for the Nutrition Unit. ICDDR,B provided the technical support by developing the capacity of staff in appropriate clinical care required for the management of severely-malnourished children through need-based training/refresher courses and developed a monitoring system to enable the health facility to measure the outcomes of the initiatives to provide evidence-base to policy-makers. Each organization was responsible for the costs associated with its contribution.

The study subjects included children, aged less than five years, from the Chittagong City Corporation and adjoining areas, who were admitted to the Nutrition Unit if their weight-for-height was <70% of the National Center for Health Statistics (NCHS) median or if they had bilateral pedal oedema or mid-upper arm circumference of <110 mm and medical complications during June 2005–May 2006. Staff recorded the medical history of the child and provided treatment as per the guidelines of WHO (6,9-11), which is briefly described below, until these requirements were met: (a) the child's condition was stable and appetite had returned, (b) absolute weight gain of ≥500 g if the child was aged less than two years, or ≥700 g if the child was aged 2-5 years, (c) for children admitted with oedema, complete loss of oedema and weight-for-height >70%, and (d) the mother or caretaker had received specific training on appropriate feeding and was motivated to follow the advice given.

### Management protocol

The children were managed in two phases: (a) acute phase and (b) nutrition rehabilitation phase. The initial treatment in the acute phase began with admission to the hospital and lasted until the child's condition was stable and appetite had returned, which usually took 3-7 days. The principal tasks during initial treatment included treatment and prevention of hypoglycaemia, hypothermia, dehydration, electrolyte imbalance, shock, and treatment of infections and other problems, including vitamin A deficiency, severe anaemia, and heart failure (6,10). Key points of acute phase management were the following:

#### Adherence to a specific feeding schedule from the beginning of treatment

The children were kept in the Nutrition Unit with their mothers/caretakers and were given locally-prepared therapeutic feed (F-75)—10 mL/kg per feed every two hours, day and night, during the initial 1-2 day(s). F-75 was a milk-based formula prepared with dried whole milk of 35 g, sugar 100 g, soybean oil 20 g, one Filwel® silver tablet [each tablet contains vitamin A 3500 IU, vitamin C 60 mg, vitamin D 400 IU, vitamin E 45 IU, vitamin K 10 mcg, thiamin 1.5 mg, riboflavin 1.7 mg, niacin 20 mg, vitamin B6 3 mg, folic acid 400 mcg, vitamin B12 25 mcg, biotin 30 mcg, pantothenic acid 10 mg, calcium 200 mg, phosphorous 48 mg, iodine 150 mcg, magnesium 100 mg, zinc 15 mg, selenium 20 mcg, copper 2 mg, manganese 2 mg, chromium 150 mcg, molybdenum 75 mcg, chloride 72 mg, potassium 80 mg, boron 150 mcg, nickel 5 mcg, silicon 2 mg, vanadium 10 mcg, lutein 250 mcg, lycopene 300 mcg (12)], and water (to make 1,000 mL) (10). Children who were very weak/unwilling to eat were fed using a nasogastric tube. After 2-3 days, the children were given three-hourly and then four-hourly feed with gradual increased amount per feed (6,10).

#### Broad-spectrum antibiotic treatment

Children without any apparent complications were given amoxicillin (45 mg/kg) orally in three divided doses. Amoxicillin or ampicillin (100 mg/kg) in four divided doses and gentamicin (7.5 mg/kg) once daily were given to children with complications, such as pneumonia, hypoglycaemia, hypothermia, severe skin lesions, or if the child appeared very lethargic and sick. In very severe cases, ceftriaxone (50-75 mg/kg) was used once daily for 5-7 days.

#### Vitamin and mineral supplementation

Vitamin A (200,000 IU) was given to children aged over one year, and 100,000 IU to infants aged 6-12 months. If the child had xerophthalmia, the same doses were repeated on day 2 and 15 or on the day of discharge. Elemental zinc was given at a dose of 2 mg/kg per day. Multivitamin drops were given twice daily: 1 mL for children aged over one year and 0.5 mL for infants aged less than one year; folic acid (1.25 mg) was given daily. These were continued throughout the nutrition rehabilitation phase. The children also received potassium chloride solution in a dose of 4 mmol potassium/kg per day three times daily for five days. These vitamins and minerals were administered separately (7,8) because the WHO-recommended (6,10) micronutrient powder—the combined mineral vitamin mix (CMV)—was not available.

#### Slower rehydration, rational use of rehydration fluids with emphasis on oral rehydration therapy

Rehydration was accomplished over a more extended period of time (8-12 hours) than the usual 3-6 hours, avoiding intravenous fluids as far as possible. Some dehydration was managed with oral rehydration solution (ORS). In severe dehydration, initial hydration was accomplished with an intravenous fluid (Na^+^, 133; K^+^, 20; Cl^−^, 98; acetate, 48 mmol/L; and 5% dextrose) until the hydration status was changed to some extent when intravenous fluid was replaced by ORS.

#### Early recognition of complications and their proper management

The common associated conditions/complications, e.g. hypoglycaemia, hypothermia, hypokalaemia, acidosis, dermatosis, heart failure, etc. were actively looked for and monitored, and treated according to the guidelines of WHO (6,10).

The initial phase of treatment ended when the child became hungry. The nutrition rehabilitation phase was then begun, during which F-100, the therapeutic diet for catch-up growth, was started. F-100 was a milk-based formula prepared with dried whole milk of 110 g, sugar 50 g, soybean oil 20 g, one Filwel® silver tablet (12), and water (to make 1,000 mL) (10). The transition was gradual to avoid the risk of heart failure. Breastfeeding was continued between formula feeds. Iron, which was not given in the initial phase, was started during the nutrition rehabilitation phase at a dose of 3 mg of elemental iron/kg per day. The other principal tasks during the nutritional rehabilitation phase were: encouraging the child to eat as much as possible, stimulating emotional and physical development, and preparing the mother or caretaker to continue to look after the child at home following discharge. A trained health worker counselled the mother/caregiver in the following areas: (a) breastfeeding and preparation of nutritious solid food with available ingredients; (b) management of fever and diarrhoea in the home; (c) danger signs of common illnesses and benefits of positive healthcare-seeking behaviours; (d) benefits of immunization and supplementation of vitamin A; and (e) use of home-made toys and simple methods for psychosocial stimulation.

### Statistical method used

Data were entered and analyzed using the SPSS software for Windows (version 10.0) (SPSS Inc, Chicago, USA) and Epi Info software (version 3.3.2). Categorical variables were compared by chi-square test, and the Fisher's exact test was applied if the expected number in any cell was 5 or less. For normally-distributed continuous variables, means were compared using 2-sample unpaired *t*-test after checking the equality of variance (Levene's test). For continuous variables not normally distributed, the Mann-Whitney U test was performed. A p value of less than 0.05 was considered to be statistically significant.

## RESULTS

During June 2005–May 2006, 171 children were admitted to the Nutrition Unit of CMCH as per the admission criteria: 130 with severe wasting (without oedema) and 41 with oedema. Table 1 describes the age, weight, and weight-for-height on admission. The number of admissions in each month ranged from 9 to 22. The highest number of admissions was observed in September 2005 (n=22) and October 2005 (n=19), just after the monsoon period (July-October). Nearly 66% of the admitted children were aged less than two years, and 84.2% belonged to households with a monthly income of less than US$ 40, suggesting insecurity of household food and nutrition status. The main reason of bringing children by families to the hospital was associated major illnesses: bronchopneumonia (33%), oedema (24%), diarrhoea (11%), pulmonary tuberculosis (9%), or other diseases, such as meningitis, septicaemia, and infection of the skin, eye, or ear.

**Table 1.** Baseline characteristics of severely-malnourished children admitted to the Nutrition Unit of Chittagong Medical College Hospital

Characteristics	All (n=171)	Severely wasted (without oedema) (n=130)	With oedema (n=41)
Age (months)	23.5±15.3	22.9±14.1	25.2±18.5
Body-weight (kg)	6.10±1.88	5.86±1.69	6. 84±2.27
Weight-for-height (% of NCHS median)	71.5±9.5	67.5±6.8	81.3±10.4

All values are of mean±SD; NCHS=National Center for Health Statistics; SD=Standard deviation

Of the 171 children admitted, the parents of 13 (7.6%) children insisted for discharging their children due to other urgent commitments while parents of 20 (11.7%) children simply absconded with their children. Of the 138 remaining children treated, 122 (88.4%) (mean±SD age 22.5±14.5 months) graduated from the Nutrition Unit following successful treatment with standardized protocol, one child was referred to another place, and 15 (10.9%) died due to associated complications: septicaemia 8, pulmonary tuberculosis 3, bronchopneumonia with haemolytic anaemia 1, aspiration of worm in airway 1, tubercular meningitis 1, and heart failure with pleural effusion 1.

The progress of nutritional rehabilitation was assessed by the rate of weight gain expressed as g/kg per day and/or disappearance of oedema. The mean gain in weight was 10.6 g/kg per day in non-oedematous children. While oedematous children had a mean weight loss of 1.9 g/kg per day, and the change of weight was observed similar in both girls and boys (p=not significant). Loss of weight was observed in 19.8%, no change in weight was observed in 3.7%, and gain in weight was observed in 76.5% of the treated children (Table 2). If the rate of gain in weight is <5 g/kg per day, the progress is considered poor; if it is 5-10 g/kg per day, it is considered moderate; if it is >10 g/kg per day, it is considered good (6,10). At the CMCH, 14.7% of the children demonstrated poor gain in weight, 30.9% moderate, and the remaining 30.9% demonstrated good gain in weight. During the early stabilization phase, i.e. during the first one or two day(s) when life-threatening problems were identified and treated, the children lost weight. Starting of gain in weight was observed during day 3-7 of the stabilization phase (especially in non-oedematous children) and during the rehabilitation phase when intensive feeding was given to recover most lost weight. During the later periods, the children showed increased appetite and major clinical improvement, including the loss of oedema.

**Table 2.** Patterns of gain in weight by age and gender (% of total)

Weight gain (g/kg/day)	% of total	6-12 months		13-24 months		25-36 months		37-60 months	
		Boys (n=14)	Girls (n=26)	Boys (n=18)	Girls (n=26)	Boys (n=13)	Girls (n=08)	Boys (n=10)	Girls (n=7)
No change in weight	3.7	7.1	0	5.6	0	0	0	10.0	0
Weight loss	19.8	7.1	26.9	5.6	3.8	23.1	37.5	40.0	14.3
Poor (<5)	14.7	14.3	0	16.7	23.1	7.7	0	30.0	42.9
Moderate (5-10)	30.9	50.0	26.9	44.4	42.3	23.1	25.0	0	28.6
Good (>10)	30.9	21.4	46.2	27.8	30.8	46.2	37.5	20.0	14.3

Table 3 shows that nearly 71% of children with oedema lost weight, and the remaining children (except one) gained weight. They took a significantly longer time to meet the exit criteria (19 days compared to 14 days taken by non-oedematous children). During the nutritional rehabilitation phase, most children with oedema did not gain weight, despite an adequate food intake. This was presumably due to oedema fluid being lost while tissue was being restored and pre-fixed discharge criteria of oedema-free weight-for-height of 70% of the reference median. Another reason of not observing much better gain in weight could be the use of locally-produced F-75 and F-100 diets. Both the diets did not contain CMV (because CMV was not available) as suggested by the WHO. No significant differences were observed in the mean gain in weight, graduation rate, and death rate between girls and boys, and children with or without oedema.

**Table 3.** Comparison of performance of children with and without oedema

Variable	Children with oedema (n=28)	Children without oedema (n=94)	p value
Change in weight			
No change (%)	3.6	2.1	NS
Loss in weight (%)	71.4	1.1	<0.001
Gain in weight (%)	25.0	96.8	<0.001
Mean±SD change in weight g/kg/day)	−1.89±5.18	10.59±6.70	<0.001
Boys	−2.09±7.05 (n=14)	9.45±6.07 (n=41)	<0.001
Girls	−1.68±2.44 (n=14)	11.47±7.09 (n=53)	<0.001
Length (days) of stay			
Mean±SD length of stay	18.8±8.8	13.6±5.9	<0.05
Boys	18.3±8.1 (n=14)	14.3±5.6 (n=41)	NS
Girls	19.3±9.7 (n=14)	13.1±6.0 (n=53)	<0.05

NS=Not significant; SD=Standard deviation

The use of the WHO guidelines for the treatment of children with severe acute malnutrition showed positive effect (Table 4) as there was an increase in the percentage of children with mild malnutrition (weight-for-height 80-90% of the NCHS median) by 24.6%. Similarly, an increase in moderate malnutrition (weight-for-height 70-80% of the NCHS median) by 4.1% was also observed. There was a 28.6% decrease in children with severe acute malnutrition (weight-for-height <70% of the NCHS median). Irrespective of the age and sex groups, a decline in the percentage of severe acute malnutrition was observed in all the children who successfully graduated (Table 4).

**Table 4.** Percentage of children with changes in nutritional status after treatment by age and sex

(Age months)	Sex	Mild (W/H 80-90%∗)		Moderate (W/H 70-80%∗)		Severe (W/H <70%∗)	
		Admission	Discharge	Admission	Discharge	Admission	Discharge
6-12	Boys (n=14)	21.4	57.1	50.0	35.7	28.6	7.1
	Girls (n=26)	19.2	65.4	46.2	19.2	34.6	15.4
13-24	Boys (n=18)	0	11.1	38.9	61.1	61.1	27.8
	Girls (n=26)	3.8	26.9	30.8	46.2	65.4	26.9
25-36	Boys (n=13)	15.4	46.2	38.5	46.2	46.2	7.7
	Girls (n=8)	25.0	50.0	37.5	37.5	37.5	12.5
37-60	Boys (n=10)	20.0	0	50.0	80.0	30.0	20.0
	Girls (n=7)	14.3	28.6	14.3	42.9	71.4	28.6
Total	122	13.1	37.7	39.3	43.4	47.5	18.9

∗Weight-for-height in percentage of the median of the National Center for Health Statistics; W/H=Weight-for-height

The average cost of overall treatment was US$ 14.6 per child or approximately US$ 1 per child-day (excluding staff-cost). Of this, cost of food was US$ 6.1 or 42%, and cost of medicines was $ 8.5 or 58% of the total cost.

## DISCUSSION

The performance of CMCH was compared with two other standards—the Sphere standard (13) and WFP/UNHCR guidelines (14) (Table 5). It was observed that 63.9% of the children graduated after meeting the exit criteria in 10-20 days while 19.7% of them graduated in less than 10 days. The length of stay for 83.6% of the children (80.0% of boys and 86.6% of girls) treated was lower than three weeks, which is consistent with the acceptable length of stay of <4 weeks recommended by the Sphere standards (13) or <3-4 weeks suggested in the WFP-UNHCR guidelines (14). Only 4% of the children stayed in the Nutrition Unit of CMCH for 31-40 days. None of the children treated in the Nutrition Unit stayed for more than 40 days. According to the WHO, a case-fatality rate (CFR) of >20% is considered to be unacceptable in the management of severe malnutrition, 11-20% is poor, 5-10% is moderate, 1-4% is good, and <1% is excellent (6,10), and according to the Sphere standards (13), management of severe malnutrition is effective when the CFR is <10%. The CFR of 10.8% (15/138) observed in the Nutrition Unit was slightly higher than the 10% cut-off of the WHO and Sphere standards. A CFR of 9% was observed in a hospital specialized in the treatment of diarrhoeal and nutritional diseases (7), and a higher (15%) CFR was reported from a tertiary-care centre in Bangladesh having facilities similar to that in the CMCH (15) and other hospitals in South Africa (16,17). Limited/delayed supply of essentials and frequent turnover of health personnel working in the severe malnutrition unit/block may affect the expected outcome in terms of gain in weight and reduction of CFR (17). The condition at the CMCH was not exceptional compared to that observed in a South African Hospital (17). Although some deaths could be prevented by eliminating the above-mentioned reasons, some deaths might not be avoidable, especially in children admitted with severe illnesses, e.g. septicaemia, severe pneumonia, etc., as also mentioned by Brewster *et al*. (18). It is worthwhile to mention that more than half (53.3%; 8/15) of fatality in children admitted to the Nutrition Unit of CMCH was due to septicaemia. A further reduced CFR may be possible in subsequent years, when the staff become accustomed with the management protocol as observed in ICDDR,B where the CFR came down from 9% to ∼5% within the next three years (Ahmed T. Personal communication, 2008). Again, the mean recovery rate at the CMCH was 88.4% which was higher than the minimum of >75% specified by the Sphere standards/WFP/UNHCR guidelines for therapeutic feeding. The mean gain in weight was 7.73 g/kg of body-weight per day in these children, which was close to the >8 g per kg/day specified in the Sphere standards/UNHCR/WFP guidelines. The mean length of stay in the Nutrition Unit to recover and exit after meeting the graduation criteria was 14.8 days which was lower than 3-4 weeks recommended by the Sphere standards/WFP/UNHCR guidelines. Finally, the default rate of 19.3% among the children treated at the CMCH was higher than <15% recommended by the Sphere standards/WFP/UNHCR guidelines. This was because more than half of the children admitted to the CMCH were from outside the Chittagong City Corporation, and nearly 11.7% of the children absconded whereas another 7.6% were discharged before they were fully recovered at the request of parents/caregivers.

**Table 5.** Comparison with indicators for monitoring therapeutic programmes

Variable	CMCH	Acceptable limits	
		Sphere standards∗	WFP/UNHCR∗
Recovery rate (%)	88.4	>75	>75
Death rate (%)	10.8	<10	<10
Mean±SD weight gain (g/kg/day)	7.73±8.30	>8	≥8
Mean±SD length of stay	14.81±6.94 (days) or 2 weeks approximately	3-4 weeks	<3-4 weeks
Defaulter (%)	19.3	<15	<15

∗Sphere standards (13); WFP/UNHCR (14); CMCH=Chittagong Medical College Hospital; SD= Standard deviation; UNHCR=United Nations High Commissioner for Refugees; WFP=World Food Programme; W/H=Weight-for-height

All parents or care providers must know how to prevent malnutrition from recurring. This was promoted through health and nutrition education to mothers during their stay at the Nutrition Unit. Planned follow-up at regular intervals after discharge is essential for ensuring the management of children in the rehabilitation phase. As the risk of relapse is the highest soon after discharge, parents were asked to bring their children back for regular follow-up (at 1, 2, and 4 weeks, then monthly for 3 months) and ensure that children received immunizations (if required) and six-monthly vitamin A capsules. However, only 43 (35%) of 122 children who graduated made a return visit for follow-up. The mean weight-for-height had increased from 76.6% to 80.8% (average 4.2%) within 20.6±12.2 days. Only eight (6.5%) children came for the second follow-up. This was not surprising as more than 50% of the children admitted were from outside the Chittagong City Corporation limits, and even for those within the Corporation limits, a visit to the hospital has financial and time implications.

Over the 12-month period, 197 health professionals were trained in managing severe acute malnutrition. Of these, 82 were medical students, 103 were medical interns, and 12 were nurses. This experience strengthened their self-confidence to manage severely-malnourished children successfully, despite working in difficult circumstances and to motivate and train other staff, leading to improved performance. Senior nursing staff of the paediatric ward were also targeted because treatment is primarily nurse-led in this type of hospitals, and they are strategically placed to identify incorrect or incomplete activities and take timely action where needed. The role of external facilitators was found to be important in the whole process. In addition to offering technical expertise and training, the external facilitators helped/supported the related medical team of CMCH during the introduction of essential changes in practices at the Nutrition Unit and gave encouragement to the paediatric staff (19).

The collaborative approach among the Nutrition Unit of CMCH, Concern Worldwide Bangladesh, and ICDDR,B has shown that within limitations in the health infrastructure, effective implementation of the WHO guidelines is feasible when the available staff members are trained and supported to follow the guidelines. Implementation leads to improved case management at par with the Sphere key indicators, as well as acceptable indicators suggested in the UNHCR/WFP guidelines.

The latest guidelines of WHO for the management of severe malnutrition should be introduced in the medical and nursing curricula of all the teaching hospitals as a first step. A nutrition unit should preferably be established in the paediatric wards of all the teaching hospitals. The objectives of establishing such a unit is not just treating severely-malnourished children, but providing essential hands-on training to nursing and medical students, nurses, and doctors. Such units can be scaled up with the help of WHO, local philanthropists, and NGOs.

The optimum mechanism of ensuring care of severely-malnourished children in the community, i.e. following discharge from the nutrition unit, is still not clear. This issue should be addressed by operations research. The engagement of local NGOs in this process should be considered.

## ACKNOWLEDGEMENTS

This study has been funded by the Government of Bangladesh through IHP-HNPRP. It was also co-funded by Concern Worldwide Bangladesh and Chittagong Medical College Hospital, Chittagong, Bangladesh. The study was supported by ICDDR,B and its donors which provide unrestricted support to the Centre for its operations and research. Current donors providing unrestricted support include: Australian Agency for International Development (AusAID), Government of the People's Republic of Bangladesh, Canadian International Development Agency (CIDA), Embassy of the Kingdom of the Netherlands (EKN), Swedish International Development Cooperation Agency (Sida), Swiss Agency for Development and Cooperation (SDC), and Department for International Development (DFID), UK. The authors gratefully acknowledge these donors for their support and commitment to the Centre's research efforts.
